# Isolation of single cells from human uterus in the third trimester of pregnancy: myometrium, decidua, amnion and chorion

**DOI:** 10.1093/oxfimm/iqac010

**Published:** 2022-11-23

**Authors:** Alexander T H Cocker, Emily M Whettlock, Brendan Browne, Pei F Lai, Jonathan K H Li, Sivatharjini P Sivarajasingam, Nesrina Imami, Mark R Johnson, Victoria Male

**Affiliations:** Department of Metabolism, Digestion and Reproduction, Imperial College London, London, UK; Department of Structural Biology, Stanford University, Stanford, CA, USA; Department of Metabolism, Digestion and Reproduction, Imperial College London, London, UK; Department of Infectious Disease, Imperial College London, London, UK; Department of Metabolism, Digestion and Reproduction, Imperial College London, London, UK; Department of Metabolism, Digestion and Reproduction, Imperial College London, London, UK; Department of Metabolism, Digestion and Reproduction, Imperial College London, London, UK; Department of Metabolism, Digestion and Reproduction, Imperial College London, London, UK; Department of Infectious Disease, Imperial College London, London, UK; Department of Metabolism, Digestion and Reproduction, Imperial College London, London, UK; Department of Metabolism, Digestion and Reproduction, Imperial College London, London, UK

**Keywords:** pregnancy, single cell, uterus, immune cells, protocol

## Abstract

During pregnancy, interactions between uterine immune cells and cells of the surrounding reproductive tissues are thought to be vital for regulating labour. The mechanism that specifically initiates spontaneous labour has not been determined, but distinct changes in uterine immune cell populations and their activation status have been observed during labour at term gestation. To understand the regulation of human labour by the immune system, the ability to isolate both immune cells and non-immune cells from the uterus is required. Here, we describe protocols developed in our laboratory to isolate single cells from uterine tissues, which preserve both immune and non-immune cell populations for further analysis. We provide detailed methods for isolating immune and non-immune cells from human myometrium, chorion, amnion and decidua, together with representative flow cytometry analysis of isolated cell populations present. The protocols can be completed in tandem and take approximately 4–5 h, resulting in single-cell suspensions that contain viable leucocytes, and non-immune cells in sufficient numbers for single-cell analysis approaches such as flow cytometry and single cell RNA sequencing (scRNAseq).

## Introduction

Human parturition is characterized by cervical ripening, myometrial contractions and rupture of the foetal membranes which is associated with the release of pro-inflammatory cytokines, ultimately leading to delivery of the infant. Although the feedback loops that drive labour once it has begun are well-established, the mechanism by which labour is initiated has not yet been definitively characterized. Doing so is an essential step in developing the most effective interventions for clinical management of preterm, term and post-term birth to improve obstetric outcomes by minimizing the risk of labour-associated complications.

One longstanding hypothesis is that an immune signal from the maternal–foetal interface induces labour although the exact role of maternal immune cells has still not been identified [1, 2]. During the first trimester, communication between immune cells in the mucosal lining of the uterus, known as the decidua, and the developing placenta is required for a successful pregnancy. Dysfunction of this relationship can result in impaired implantation, which can lead to complications such as miscarriage, foetal growth restriction and pre-eclampsia [3–6]. The importance of these interactions has led to the development of robust laboratory protocols for isolating immune and non-immune cells from first-trimester tissues [7], and these tissues have now been very extensively phenotyped [8, 9].

The immune cell environment, at least in the decidua, is dynamic throughout pregnancy [10, 11]. Therefore, findings from first-trimester tissues cannot be used to answer the questions about the third trimester. So far, many studies have observed changes in immune cell populations in the decidua during term and preterm labour [12–14], including one which suggests that decidual immune changes precede labour [2]. Macrophages increase in number in the decidua parietalis during labour, and evidence from rat models suggests that this infiltration precedes labour [2, 15]. There is also evidence from other tissue-based studies, including a uterine overdistention study in non-human primates [16], that cytokine levels increase in the amniotic fluid prior to labour. However, there is still much that is unknown about the phenotype and function of these immune cell infiltrations and the mechanism by which they could contribute to the initiation of myometrial contractions that are necessary for labour to proceed.

The decidua lies between the myometrium and the chorionic layer of the foetal membranes (decidua parietalis) and placenta (decidua basalis), and directly interacts with both tissues to influence function. Previous integrative analysis of three bulk transcriptomics datasets identified 126 differentially expressed genes in the myometrium by comparison of biopsies from non-labouring and labouring women at term gestation [17]; tumour necrosis factor alpha (TNFα) signalling, cytokine receptor signalling, Jak-STAT signalling, NOD-like nucleotide-binding oligomerization domain-like (NOD-like) receptor signalling and chemokine signalling pathways were all highlighted by pathway enrichment analysis as significant contributors to the process of labour [17]. TNFα, along with other cytokines, has also been identified as differentially expressed during term labour in uterine tissues by a systematic review of 172 studies [18]. However, none of these studies have explored individual cell populations to determine the source of these signals, nor the extent to which they propagate across the uterus.

**Figure 1.**
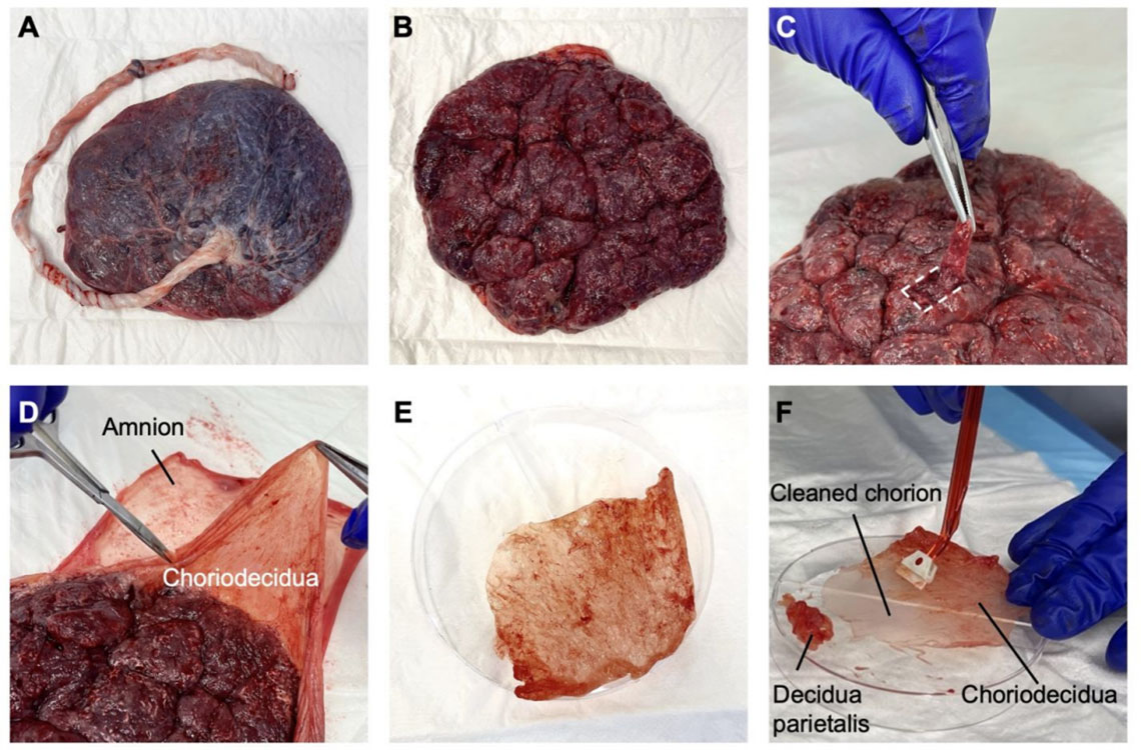
Anatomy and dissection of the decidua and foetal membranes. (**A**) The amniotic surface (foetal side) of placenta with umbilical cord visible; amnion covers the placental tissue. (**B**) The basal plate of placenta; a thin layer of grey/silver decidua basalis is visible. (**C**) Dissection of decidua basalis from the basal plate. In order to lift the flap of decidua, as shown, it has first been cut on three sides, and then gently cut away from the underlying trophoblast using scissors. (**D**) The amnion (in the background of the image) has been peeled away from the choriodecidua (in front of the amnion in the image). The decidua parietalis is identified on the maternal surface of the chorion. Sections of choriodecidua are cut away for further dissection using scissors. (**E**) Choriodecidua with decidua parietalis facing upwards; decidua parietalis is a soft, loosely aggregated tissue with reticulated pink/red pattern. (**F**) The decidua parietalis is scraped off the chorion.

To understand uterine regulation of labour onset and progression, research on both the phenotype and function of individual immune and non-immune cell subsets in key reproductive tissues (myometrium, decidua, chorion, amnion and placenta; Fig. 1) is needed. To enable this, we developed protocols to isolate immune and non-immune cells from tissues into suspensions suitable for single cell-based experiments. To aid researchers using these protocols, we have also included representative results following the extraction of cells from these tissues, which form a guide to expected cell viability, frequencies and overall yield.

## Protocol 1: isolation of myometrium cells

Myometrial tissue is predominantly composed of smooth muscle cells, which require harsh disaggregation to separate from the extracellular matrix (as is the case for other non-immune cells; e.g. endothelial cells and fibroblasts) [19]. This process can destroy relatively more delicate cell types present, such as immune cells [19]. Protocol 1 starts with gentle dissociation and ends with harsher conditions for single-cell extraction from myometrial tissue, which we demonstrate can extract viable immune and non-immune cells. The protocol has an overall duration of ∼3–4 h. When samples are processed immediately after biopsy excision, resulting cell viability of >95% is achievable based on Trypan blue staining. Note that we specify the use of lower segment myometrium biopsies because this is what was used for protocol development; this protocol can be adapted for upper segment myometrium biopsies.

### Materials for myometrium tissue disaggregation (single cells isolation)

#### Equipment, sterile-packed plasticware and single-use items

- GentleMACS Dissociator (Miltenyi Biotec).
- Shaking water bath set at 37°C and 100 rpm.
- Centrifuge with swing bucket rotor for 50-ml tubes and adjustable acceleration/deceleration.
- Cell counter (haemocytometer or automated; former needs light microscope too).
- Electronic balance.
- Watchmaker forceps.
- Disposable scalpels (blade No. 22).
- 10-cm diameter Petri dishes
- 0.45-µm syringe filter.
- GentleMACS C-tube (Miltenyi Biotec 130-096-334).
- 70-µm cell strainers.
- 50-ml centrifuge tubes.
- 3-ml Pasteur pipettes.
- 20- and 10-ml syringes.
- Parafilm.
- Pipette controller.
- Serological pipettes (5-, 10- and 25-ml capacity).
- Micropipettes.
- 10-µl and 1-ml micropipette tips.

#### Reagents

- Collagenase IA (Sigma-Aldrich C9891).
- Collagenase XI (Sigma-Aldrich C9407).
- Accutase (Invitrogen 00-4555-56)—store in 5-ml aliquots (using 15-ml centrifuge tubes) at −20°C.
- Dulbecco’s Modified Eagle Medium (DMEM, high glucose; Gibco 31966047).
- DMEM/Nutrient Mixture F12 (Gibco 31331093).
- 7.5% w/v bovine serum albumin (BSA) solution (Sigma-Aldrich A8412).
- Dulbecco’s phosphate-buffered saline (DPBS, Ca^2+^ and Mg^2+^ included; Sigma-Aldrich D8662)—store in 15-ml aliquots (using 30-ml Universal tubes) at 2–4°C for biopsy collections.
- Ca^2+^ Mg^2+^-free DPBS (DPBS without Ca^2+^ and Mg^2+^; Gibco 14190169).
- ACK (Ammonium-Chloride-Potassium erythrocyte lysis) buffer (Gibco A1049201).
- Trypan blue (Gibco 15250-061).
- Virkon or similar laboratory disinfectant.
- 70% v/v ethanol.
- Wet ice.

### Myometrium tissue disaggregation protocol

#### Before you start

- Pre-heat the shaking water bath to 37°C.
- Prepare Virkon (or similar) disinfectant solution for clean-up of biological spills and waste decontamination prior to disposal. We used four Virkon tablets to 1 l of water to give final Virkon concentration of 1% when waste fluid is added to a total of 2 l.
- Wipe down work surfaces to be used for tissue preparation with 70% v/v ethanol.
- Prepare the following reagents:
  - Accutase: defrost a sterile 5-ml aliquot at room temperature.
  - Collagenase/BSA mixture: Weigh 0.01 g of both types of collagenases IA and XI into a 50-ml centrifuge tube. Dissolve the collagenases by adding 5-ml 7.5% w/v BSA solution, 10-ml DMEM (high glucose) and 10-ml DMEM/Nutrient Mixture F12. Secure the cap and place it in the 37°C shaking water bath for 5–10 min; this can be done at the same time as the first step disaggregation of tissues. Filter the collagenase/BSA solution using a 20-ml syringe fitted with a 0.45-µm syringe filter into a new 50-ml centrifuge tube. Add an additional 10-ml DMEM (high glucose) and 10-ml DMEM/Nutrient Mixture F12 to the collagenase/BSA mixture to give a final working volume of 45 ml.
  - DPBS (Ca^2+^ and Mg^2+^ included): Prepare a sterile 15-ml aliquot (chilled at 2–4°C) for biopsy collection.
  - Ca^2+^ Mg^2+^-free DPBS: Aliquot 20 ml into a 50-ml centrifuge tube, secure its cap and weigh. Record the weight (to be used later to determine biopsy weight) and maintain it at room temperature.

#### Procedure

I. Acquisition of lower segment myometrium biopsy at caesarean section:
  1. After delivery of the foetus and placenta, a professional with relevant surgical qualifications cuts a myometrial biopsy from the upper edge of the caesarean incision made in the lower uterine segment using surgical scissors.
  2. Immediately immerse the biopsy in the 15-ml DPBS aliquot and secure the cap to transport on wet ice from the operating theatre to the laboratory.
  3. Commence tissue preparation (i.e. dissection and dissociation) within 1 h of biopsy excision from the uterus. Alternatively, keep the biopsy in DPBS to temporarily store at 2–4°C until ready to proceed with isolation of cells.
    - Note: Tissue quality will deteriorate over time even when temporarily stored at 2–4°C, which will reduce the number of viable cells to be isolated.
II. Tissue preparation:
  4. Transfer 2–5 g myometrial tissue into the 20-ml Ca^2+^ Mg^2+^-free DPBS aliquot, weigh and calculate the exact tissue weight. Transfer the biopsy with DPBS into a Petri dish to finely dissect into ∼1 mm cubes using sterile scalpels (with Watchmaker forceps to hold down large pieces while dissecting) until the minced tissue can be drawn up into a Pasteur pipette without blocking.
    - Note: Myometrium biopsies are vascularized, and some biopsies may contain scar tissue, cervical tissue, decidua and blood clots. These should be dissected out prior to mincing the remaining myometrial smooth muscle to minimize contamination of irrelevant cell types and aid subsequent disaggregation steps.
    - Note: Cutting the tissue into ∼1 mm pieces improves yield by increasing surface area for contact with accutase and collagenases.
    - Note: Accutase requires a Ca^2+^ Mg^2+^-free environment for optimal enzymatic activity.
  5. Transfer the minced tissue into a 50-ml tube. Securely cap the tube to centrifuge at 400*g* for 5 min at room temperature, with brake and acceleration on the maximum setting.
III. First step disaggregation:
  6. Aspirate the supernatant from the pellet of minced tissue and resuspend it in 5-ml accutase. Securely cap the tube, wrap Parafilm around the cap and horizontally immerse the tube in the 37°C shaking water bath to incubate for 15 min.
    - Note: The parafilm helps to prevent water from the bath seeping into the tube, which would contaminate the tissue.
    - Note: Holding the tube in the water bath in a horizontal position maximizes surface area contact by accutase during agitation by shaking. Use water-resistant tape to secure the tube to either the inner wall of the water bath or the side of a tube rack.
  7. After the 15-min incubation, retrieve the tube of accutase-treated tissue from the water bath. Wipe off the water and clean the tube’s external surfaces with 70% v/v ethanol prior to removing the cap to strain the tissue suspension through a 70-µm cell strainer attached to a new 50-ml centrifuge tube.
  8. Once the supernatant (cell suspension) stops passively flowing through the 70-µm cell strainer, use the plunger of a 10-ml syringe to gently press down on the solid tissue trapped in the cell strainer to force any remaining supernatant to combine with the rest of the flow through in the attached 50-ml tubes.
    - Note: Multiple cell strainers may be required to retrieve the cell suspension. If the initial cell strainer blocks, pour the solid material in the blocked strainer into a new 70-µm cell strainer and repeat the process to increase yield.
    - Note: Do not discard the solid tissue. This will be used for the second step disaggregation.
  9. Wash the cell strainer(s) with Ca^2+^ Mg^2+^-free DPBS until a total volume of 40-ml cell suspension is obtained.
  10. Securely cap the tube of the cell suspension to centrifuge at 400*g* for 5 min at room temperature, with brake and acceleration on the maximum setting.
    - During this centrifugation step: commence second step disaggregation of tissue retained in the 70-µm cell strainer(s)—go to section IV (Steps 15 and 16).
  11. Aspirate the supernatant from the cell pellet and resuspend it in ACK buffer (1 ml/2 g tissue). Incubate the cells with ACK buffer for 5 min at room temperature.
  12. Add DPBS to the cells in ACK buffer to obtain a final volume of 40 ml. Securely cap the tube to centrifuge at 400*g* for 5 min at room temperature, with brake and acceleration on the maximum setting.
  13. Aspirate the supernatant from the cell pellet and resuspend it with 1-ml Ca^2+^ Mg^2+^-negative DPBS.
  14. Mix a 10-µl sample of this final cell suspension with Trypan blue for cell counting (or alternative dye if using an automated cell counter). Record count of live and dead cells; calculate percent viability. Proceed to experiments with the remaining cells as required (once cells from the second step disaggregation are also ready).
IV. Second step disaggregation
  15. Use a Pasteur pipette to transfer the solid tissue trapped in 70-µm cell strainer(s) from the first step disaggregation into a gentleMACS C-tube and add 10-ml collagenase/BSA mixture.
  16. Attach the C-tube of tissue to the gentleMACS dissociator and select the present ‘(Mouse) Neonatal Heart’ (NH) programme (‘m_neo_heart_01.01’; 37 s × 1 cycle) and proceed with mechanical dissociation.
  17. After the programme run has been completed, carefully remove the C-tube without opening the cap, which needs to be tightened and sealed with Parafilm for 30-min incubation in the 37°C shaking water bath. Attach the tube to the water bath as done for the first step disaggregation (Step 6).
  18. After the 30-min incubation, retrieve the C-tube of collagenase-treated tissue from the water bath. Wipe off the water and clean the tube’s external surfaces with 70% v/v ethanol before removing the cap to strain the tissue suspension through a 70-µm cell strainer attached to a new 50-ml centrifuge tube.
  19. Retrieve cell suspension from 70-µm cell strainer(s), make volume up to 40 ml with DPBS, centrifuge at 400*g* for 5 min at room temperature, aspirate supernatant and resuspend the cell pellet with ACK buffer, repeat centrifugation, aspirate supernatant and resuspend with DPBS, and undertake cell counting in the same way as described for first step disaggregation (section III: Steps 8–14).
    - Note: Use DPBS that contains Ca^2+^ Mg^2+^ to maintain the viability of the smooth muscle cells.

## Protocol 2: Isolation of decidual, amnion and chorion cells

Whilst myometrium is dense muscle tissue, the decidua, amnion and chorion are less solid in their tissue structure. Nevertheless, these tissues also require enzymatic and mechanical disaggregation to recover cells. Protocol 2 extracts both leucocyte and non-immune cells from decidua basalis, decidual parietalis, amnion and chorion with high viability and yield for downstream experiments.

### Materials for decidua, amnion and chorion disaggregation (single cells isolation)

#### Equipment, sterile-packed plasticware and single-use items

- GentleMACS Dissociator (Miltenyi Biotec).
- Shaking water bath set at 37°C and 100 rpm.
- Magnetic stirring plate.
- Magnetic stirrer (flea).
- Dissection scissors.
- Dissection forceps.
- Disposable scalpels (blade No. 22).
- 10-cm diameter Petri dishes.
- 300-ml glass beaker (autoclaved).
- GentleMACS C-tube (Miltenyi Biotec 130-096-334).
- 50-ml centrifuge tubes.
- 70-µm cell strainers.
- 3-ml Pasteur pipettes.
- Pipette controller.
- Serological pipettes (5-, 10- and 25-ml capacity).
- 10-ml syringes.
- Parafilm.
- Cell scraper (Sarstedt 83.3950).
- Disposable absorbent pads (Medisave 55525).
- Paper towels.

#### Reagents

- Accutase (Invitrogen 00-4555-56)—store in 5-ml aliquots at −20°C.
- Histopaque-1077 (Sigma-Aldrich 10771).
- Foetal calf serum (FCS; Sigma Aldrich F9665).
- 0.5 M EDTA (Invitrogen 15575020).
- Ca^2+^ Mg^2+^-free DPBS (DPBS without Ca^2+^ and Mg^2+^; Gibco 14190169).
- ACK (erythrocytes lysis) buffer (Gibco A1049201).
- Trypan blue (Gibco 15250-061).
- Virkon or similar laboratory disinfectant.
- 70% v/v ethanol.

### Decidua, amnion and chorion tissue disaggregation protocol

#### Before you start

- Pre-heat the shaking water bath to 37°C.
- Prepare Virkon (or similar) disinfectant solution for clean-up of biological spills and waste decontamination prior to disposal. We used four Virkon tablets to 1 l of water to give final Virkon concentration of 1% when waste fluid is added to a total of 2 l.
- Wipe down work surfaces to be used for tissue preparation with 70% v/v ethanol.
- Prepare the following reagents:
  - Accutase: defrost a sterile 5-ml aliquot at room temperature.
  - Wash buffer: Supplement 500-ml Mg^2+^ Ca^2+^-free PBS with 5-ml FCS (equating to 1% v/v) and 2-ml 0.5 M EDTA (equating to 2 mM). Aliquot ∼200 ml to use for the day and store the rest at 2–4°C. The remaining wash buffer should be used within 1 month.
  - Histopaque-1077: Aliquot 20 ml into each of 4 × 50 ml centrifuge tubes. Maintain at room temperature.
  - Ca^2+^ Mg^2+^-free DPBS: Aliquot 20 ml into a 50-ml centrifuge tube (prepare one tube for each tissue type), secure its cap and weigh. Record the weights (to be used later to determine dissected tissue weight) and maintain all these tubes at room temperature.
    - Note: All DPBS wash steps use Ca^2+^ Mg^2+^-free DPBS in Protocol 2.

#### Procedure

I. Collection of placenta with foetal membranes:
  1. Immediate processing is recommended, ideally within 45 min after extraction from the uterus.
    - Note: Sample quality will deteriorate over time even if temporarily stored at 4°C. A cut-off duration of 45 min was chosen for our protocol development, but this can be further optimized to meet specific needs of downstream experiments that require different levels of cell viability and population numbers described by our representative data.
  2. Place two disposable absorbent pads on the tissue dissection bench. Place the placenta onto the top absorbent pad with the foetal side (where the umbilical cord protrudes; Fig. 1A) facing down onto the pad to soak up blood (and DPBS to be used for washing tissues).
    - Note: The membranes may have meconium on them, which is visible as a green-brown substance. This needs to be either avoided or washed off the membranes. It should not be included in the samples to be used for the isolation of cells.
II. Tissue dissection—Decidua basalis:As shown in Fig. 1B, the decidua basalis lies on the maternal side of the placenta, which is on the opposite side of the umbilical cord. It has a greyish colour, distinguishing it from the underlying red placental tissue.
  3. Use paper towels to remove excess blood from the placenta (maternal side).
  4. Use tissue dissection forceps to pierce and hold up the decidua (thin greyish layer on the maternal side of placenta), then use dissection scissors to cut the decidua away from the underlying placental tissue without disrupting the decidua (Fig. 1C).
  5. Transfer one tissue piece into the Petri dish with the decidua (grey and smooth) facing downwards and the underlying placental villi (pink and ruffled) facing up. Add DPBS to hydrate the tissue. Use the forceps to hold down tissue and cut away villous tissue and blood clots using the scissors.
  6. Place dissected decidua basalis (cleared of placental villi) tissue into a pre-weighed 50-ml tube of 20-ml DPBS.
  7. Repeat Steps 4–6 until sufficient decidua basalis has been cleaned. The DPBS in the Petri dish will need to be changed regularly to minimize accumulation of blood.
    - Note: This step reduces the potential for contamination from peripheral blood. However, leucocytes originating from blood cannot be eliminated entirely. Using known markers specific to tissue-resident leucocytes [9] and including peripheral blood mononuclear cell (PBMC) samples as controls in assays are ways to assess and control for any cells originating from blood.
    - Note: We routinely achieve a yield of 3–6 million cells per gram of cleaned decidua basalis. New users of the protocol can use this to estimate how much decidua basalis they should dissect to achieve their required number of cells.
  8. Transfer tissue into an autoclaved beaker with a magnetic stirrer and fill with ∼150-ml DPBS. Place the beaker onto a magnetic stirring plate.
  9. Set the stirring speed to half the maximal for a few seconds to lift all the tissue into movement, and then reduce to the lowest speed for gentle uniform agitation. The tissue should be moving around at a consistent speed but not so fast that the tissue might be damaged.
  10. Stir for 20 min total, carefully aspirating the DPBS to replace it with fresh DPBS after the first 5 min and at the end of the remaining 15 min.
    - Note: Placentas are highly vascularized so cutting into the decidua basalis can cause a large bleed that will obscure the dissection area. Soak up the blood carefully with paper towels multiple times until the tissue can be seen clearly enough to isolate the decidua basalis.
    - Note: Some placentas can have large areas of calcification. It is best to avoid these areas. If there are small amounts of calcification all over the placenta, try to avoid these as best as possible and remove any remnants at Step 5.
III. Tissue dissection—Amnion:The membrane sac attached to the placenta is composed of three layers. With the maternal side of the placenta facing upwards, the uppermost layer (which would be furthest from the foetus during pregnancy) is decidua parietalis. Underlying the decidua parietalis is the foetal chorion, and the bottom layer (which would be closest to the foetus during pregnancy) is the amnion (Fig. 1D).
  11. Using gloved hands or two pairs of dissection forceps, grip the amnion and peel it away from the other two layers: it should peel away easily as one thin smooth translucent layer.
    - Note: If the interface between the amnion and chorion is not visible to aid their separation, it sometimes helps to make a small cut/tear at the edge of the foetal membranes to expose this interface (similar to opening a plastic bag).
  12. Using dissection scissors, cut the amnion away from the rest of the placenta and then cut it further into ∼8 × 8 cm pieces.
  13. Place each 8 × 8 cm piece of amnion on top of an inverted Petri dish to cut again into smaller (∼1 × 1 cm) pieces using a scalpel.
    - Note: Cutting up the amnion into small ∼1 × 1 cm pieces is time-consuming but necessary as it increases surface area for enzymatic digestion to significantly improve the yield of single cells to be isolated.
  14. Transfer the dissected amnion tissue pieces into a pre-weighed 50-ml tube of 20-ml DPBS. Weigh the tube and calculate the tissue weight before proceeding to disaggregation (section VI).
IV. Tissue dissection—Decidua Parietalis:
  15. Cut the remaining chorionic membrane with decidua parietalis layer attached (i.e. choriodecidua) into 10 × 5 cm pieces.
  16. Place each 10 × 5 cm piece of choriodecidua membrane on top of an inverted Petri dish (Fig. 1E), rinse with DPBS and use a cell scraper to isolate the decidua parietalis layer from the chorionic membrane; the latter should be visible on the Petri dish as an intact smooth translucent membrane (similar in appearance to the amnion) if the decidua parietalis has been scraped off sufficiently (Fig. 1F).
  17. Transfer the decidua parietalis tissue (without chorionic membrane) into a pre-weighed 50-ml tube of 20-ml DPBS. Weigh the tube and calculate the tissue weight before proceeding to disaggregation (section VI).
    - Note: The decidua parietalis may have small blood clots on the surface. These should be removed with forceps before scraping the tissue.
    - Note: Scraping with the correct pressure to remove the decidua parietalis but leaving the chorion requires experience. As a general rule, if the tissue that is scraped off seems too liquid to pick up with forceps, insufficient pressure has been applied. If the underlying chorion tears, then too much pressure has been applied.
V. Tissue dissection—Chorion:
  18. Once all the decidua parietalis has been scraped off, use a scalpel to cut the chorion tissue that remains on the inverted Petri dish into small ∼1 × 1 cm pieces.
  19. Transfer the dissected chorion tissue pieces into a pre-weighed 50-ml tube of 20-ml DPBS. Weigh the tube and calculate the tissue weight before proceeding to disaggregation (section VI).
    - Note: Cutting up the chorion into small ∼1 × 1 cm pieces is time-consuming but necessary as it increases surface area for enzymatic digestion to significantly improve the yield of single cells to be isolated.
VI. Mechanical disaggregation and enzymatic digestion:
  20. Centrifuge tissues in their individual tubes of 20-ml DPBS at 400*g* for 5 min at room temperature.
  21. Carefully aspirate supernatants and re-suspend one part weight (g) of tissue to two parts volume (ml) of accutase (e.g. 5 g tissue = 10-ml accutase).
    - Note: The supernatant cannot be poured off the pellet because the tissue will be loosely packed together; use a serological pipette for aspiration to avoid loss of tissue.
  22. Transfer each tissue–accutase suspension into its own C-tube and cap securely.
  23. Attach each C-tube of tissue to the gentleMACS dissociator and select the preset ‘Mouse Spleen’ programme (‘m_spleen_01.01’; 56 s × 1 cycle) to proceed with mechanical dissociation.
  24. After the programme run has been completed, carefully remove the C-tube and loosen any tissue that may be caught on the inner side of the cap to resuspend them.
  25. Securely cap the tube, wrap Parafilm around the cap and horizontally immerse the tube in the 37°C shaking water bath to incubate for 45 min.
    - Note: The parafilm helps to prevent water from the bath seeping into the tube, which would contaminate the tissue.
    - Note: Holding the tube in the water bath in a horizontal position maximizes surface area contact by accutase during the agitation by shaking. Use water-resistant tape to secure the tube to either the inner wall of the water bath or the side of a tube rack.
  26. For each of the decidua basalis, decidua parietalis, amnion and chorion, prepare a new 50-ml tube for the capture of the cell suspension from the digested tissues by attaching a 70-µm cell strainer.
  27. Transfer the digested tissues each into their assigned 70-µm cell strainer and push the supernatant (cell suspension) through the pores using the plunger from a 10-ml syringe.
    - Note: Add the tissue a little bit at a time to the strainer to ensure tissue does not spill over when using the plunger to encourage flow through of the supernatant.
    - Note: If the strainer becomes blocked, transfer the solid tissue into a new 70-µm strainer to maximize the yield of the supernatant.
    - Note: If no more supernatant is passively flowing through and the strainer is not blocked, the tissue can be removed from the strainer and disposed.
    - Note: Small amounts of DPBS can be added to wash the C-tubes for maximal retrieval of digested tissues to place into the strainer. However, the total volume of supernatant to be captured as flow through from the strainers should not exceed 20 ml per sample.
  28. Once all supernatant has been captured from the strainers, discard the strainers and (if necessary) add DPBS until the final volume for each cell suspension equates to 20 ml.
  29. For each sample, uncap a 20-ml aliquot of Histopaque-1077, hold the tube at a 45-degree angle, and use a 10-ml serological pipette to layer the cell suspension on top of the Histopaque-1077 using the slowest setting of the pipette controller.
    - Note: Pipette onto the side of the centrifuge tube 2–3 cm above the Histopaque-1077 layer to prevent mixing of this density gradient medium with the cell suspension; although tissue-derived cells for this protocol tend to be less prone to such mixing at the interface prior to centrifugation than peripheral blood samples.
  30. Carefully cap each tube of cell suspension layered onto Histopaque-1077 to centrifuge at 450*g* for 20 min at room temperature. Important: use the slowest centrifuge acceleration setting and select the ‘no brake’ for deceleration.
  31. After centrifugation for Histopaque layering, use a Pasteur pipette to retrieve cells for each sample from the interface fraction (between the DPBS and Histopaque-1077) and transfer into a new 50-ml centrifuge tube until the boundary between the DPBS and Histopaque transitions from a cloudy to sharp appearance. This typically equates to around 10 ml.
    - Note: The interface fraction will not be as clear to the eye as when Histopaque is used to isolate PBMCs from peripheral blood. The interface may appear as only a slight blurring between the medium and Histopaque medium, but it will appear more distinct when all of the interface fraction has been removed.
  32. Once all of the interface fraction has been retrieved for each sample, add Wash Buffer until the final volume for each cell suspension equates to 40 ml
  33. Centrifuging the cell suspension in Wash Buffer at 700*g* for 10 min at room temperature with high acceleration and brake settings.
  34. Following centrifugation, discard the supernatant and re-suspend the cell pellet in 5-ml Wash Buffer.
  35. Mix a 10-µl sample of this final cell suspension with Trypan blue for cell counting (or an alternative dye if using an automated cell counter). Record count of live and dead cells; calculate percent viability. Proceed to experiments with the remaining cells as required.

### Representative results

#### Sample details and ethics

We optimized this protocol using samples obtained from healthy term pregnancies during elective/planned caesarean sections after informed written consent was provided by study participants in accordance with the Declaration of Helsinki guidelines. Samples were collected as part of the preterm labour of known and unknown aetiologies vs term labour (PREMS) study (approved by the Brompton and Harefield Research Ethics Committee 10/H0801/45) or under project R22010 of the Imperial College Healthcare Tissue Bank (approved by REC3 Wales 17/WA/0161). All samples were collected at Chelsea and Westminster hospital (London, UK).

#### Timing for the protocols

**Figure 2.**
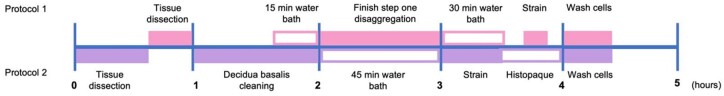
Graphical timeline for running Protocol 1 (myometrium) and Protocol 2 (decidua and foetal membranes) in parallel. Filled blocks represent active times in the protocol, for example dissection, and empty blocks represent passive times, for example the sample has an incubation in a water bath. The total process should take 4–5 h depending on user experience and the amount of tissue collected during the dissection stages.

It is best to start processing with the decidua basalis so that the tissue can be on the magnetic stirrer whilst the other tissues are being processed (myometrium and membranes; Fig. 2). Once the decidua basalis has been cleaned, all tissues can be centrifuged for 5 min at 400*g* and resuspended in accutase. The myometrium will be placed in the water bath in a centrifuge tube for 15 min, while the tissues for Protocol 2 are mechanically dissociated using the gentleMACS system. Protocol 2 tissues will then be in the water bath for 45 min, during which Protocol 1 can be continued, once the 15-min myometrium incubation is complete. After the 45-min water bath incubation (Protocol 2) is complete, the first step of myometrium disaggregation should also be finished and the second step of myometrium disaggregation (Protocol 1) will have started. Thus, at this point, the decidua, amnion and chorion samples can be taken out of the water bath and replaced with the myometrial tissue for its 30-min incubation. Whilst the tissues for Protocol 2 are centrifuged after layering onto Histopaque, the 30-min incubation for myometrium (Protocol 1) should be complete and the cell suspension from this tissue can be isolated using the cell strainer(s), washed and erythrocytes lysed. When the Histopaque interface fraction is collected from tissues for Protocol 2, all tissues for both protocols can be centrifuged together at 700*g* for 5 min in preparation for cell counting.

#### Flow cytometry for assessing isolated single cells

Cells isolated from uterine tissue samples (1 × 10^6^) were stained in 145-µl volumes in the dark at room temperature for 20 min with the following titrated fluorescently labelled monoclonal antibodies: CD103 (1.5 µl; Ber-ACT8, BV786; Biolegend), CD56 (2 µl; NCAM16.2, BV650; BD Biosciences), CD127 (5 µl; HIL-7R-M21, BV421; BD Biosciences), Aqua dead cell dye (BV510, ThermoFisher), CD45 (20 µl; HI30, FITC; BD Biosciences), CD3 (3 µl; SK7, PerCP-Cy5.5; BD Biosciences), CD31 (20 µl; WM59, PE; BD Biosciences), CD14 (2 µl; TÜK4, PE-CF594; Miltenyi Biotec), CD16 (3 µl; 3G8, PE-Cy7; BD Biosciences), CD25 (2 µl; 4E3, APC; Miltenyi Biotec), CD15 (5 µl; SSEA-1, Alexa Fluor 700; Biolegend) and CD90 (2 µl; REA897, APC-H7; Miltenyi Biotec). Stained cells were acquired immediately on a BD Fortessa X20 cytometer.

**Figure 3.**
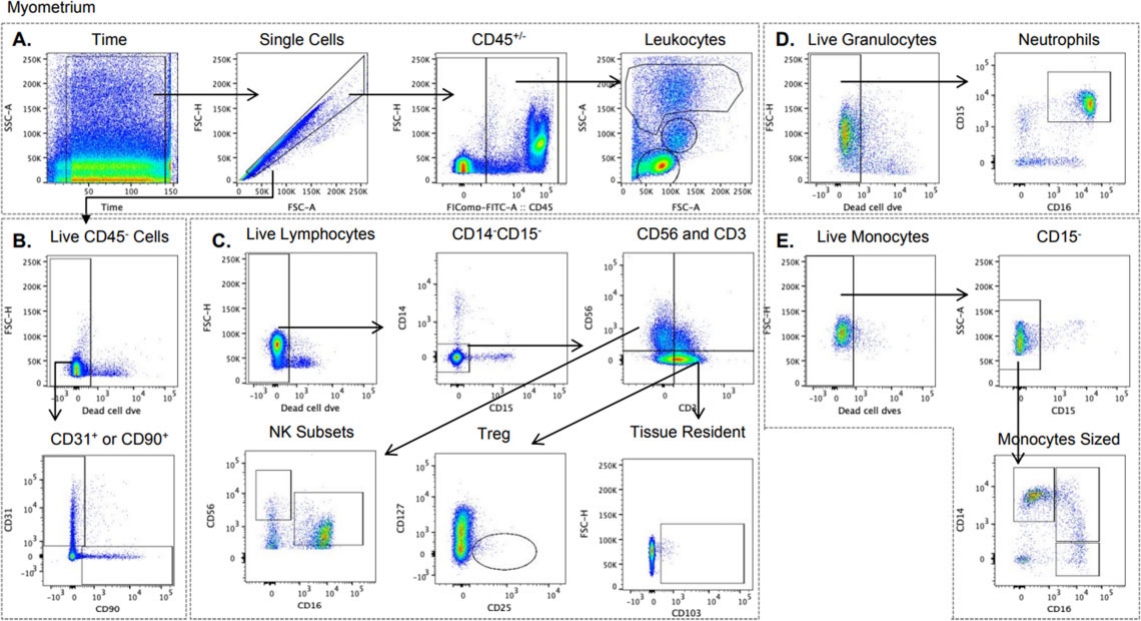
Flow cytometry gating strategy of myometrium tissue used to determine cell populations and frequencies present in single-cell isolations. (A) Identification of single cells, CD45^+/−^ cells and CD45^+^ gates for granulocytes, monocytes and lymphocyte subsets based on their size and granularity. (B) Gating of live CD45^−^ cells, then identification of CD31^+^ (endothelial) or CD90^+^ (fibroblast) cells. (C) Gating of live lymphocytes, exclusion of CD14^+^ and CD15^+^ cells, identification of CD3^+^ T cells, tissue-resident T cells (CD103^+^), and regulatory T cells (CD127^low^CD25^+^), and both CD56^bright^ (CD56^high^CD16^−^) and CD56^+^CD16^+^ NK cell subsets. (D) Gating of live granulocytes and identification of neutrophils as CD15^+^CD16^+^. (E) Gating of live monocyte-sized CD15^−^ cells as CD14^+^CD16^−^, CD14^+^CD16^+^ and CD14^−^ CD16^+^ subsets. Gates were set using both unstained and fluorescence minus one controls.

Gates were set on Flowjo v10.8.1 using unstained and fluorescent minus one controls (Fig. 3 and Supplementary Figs 2–6). Single cells were divided by CD45 expression, and CD45^−^ cells were defined as endothelial (CD31^+^) or fibroblast (CD90^+^) cells. CD45^+^ cells were defined as granulocytes, monocytes or lymphocytes based on size and granularity. CD15^+^CD16^+^ live granulocyte cells were defined as neutrophils. Live monocyte-sized CD15^−^ cells were defined as CD14^+^CD16^−^, CD14^+^CD16^+^ or CD14^−^ CD16^+^. CD14^−^ CD15^−^ live lymphocyte cells were defined as T cells (CD3^+^CD56^−^) or natural killer (NK) cells (CD3^−^ CD56^+^). Tissue-resident (CD103^+^) and regulatory (CD127^−^ CD25^+^) populations were identified from the T-cell gate. CD56bright (CD56^high^CD16^−^) and CD56^+^CD16^+^ subsets were defined in the NK gate.

#### Statistical analyses of representative data

Non-parametric tests were used as no assumptions about data distribution were made. Wilcoxon tests were used for paired data comparison, and Kruskal–Wallis tests, corrected for multiple comparisons using the Benjamini, Krieger and Yekutieli false discovery rate method, were used to compare between tissue data. Prism v9.3.1 (GraphPad) was used for data analysis and visualization.

Cells isolated from myometrium tissue were stained with the above panel of fluorescent monoclonal antibodies and acquired immediately on a BD Fortessa X20 cytometer. Representative staining of the other tissues (amnion, chorion, decidua basalis and decidua parietalis) is shown in Supplementary Figs 1–4. Samples were checked for any disrupted acquisition using a time gate, and any periods were excluded prior to further gating.

### Optimization of mechanical and enzymatic disaggregation of myometrial biopsies

**Figure 4.**
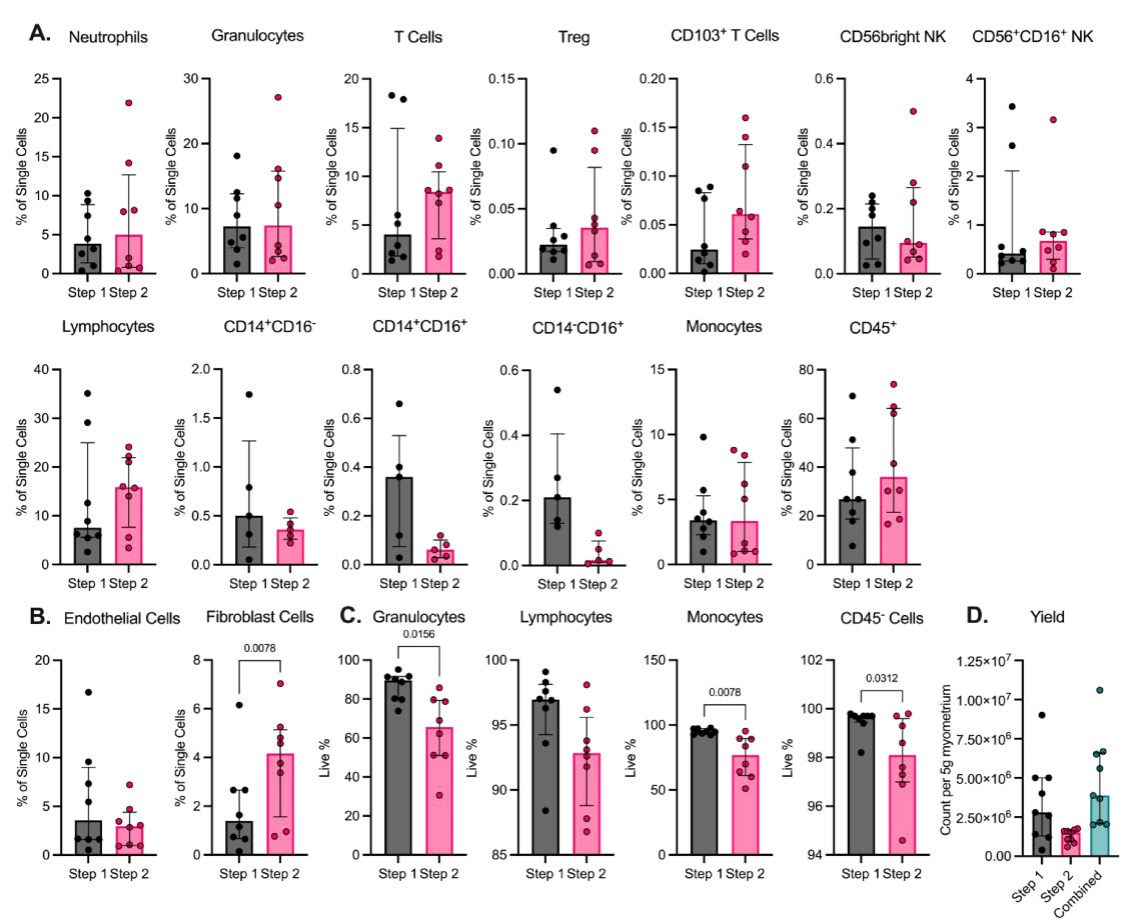
Comparison of cells recovered after the accutase incubation (Step 1) and those that underwent mechanical disaggregation with collagenases (Step 2; N = 8). (A) Frequency of immune cell subsets within the single-cell gate. (B) Frequency of endothelial and fibroblast cells within the single-cell gate. (C) Proportion of live cells in leucocyte and CD45− cell populations. (D) Recovered cell counts per 5-g myometrium (N = 9). For (A–C), paired non-parametric Wilcoxon tests were used for statistical analyses and significance defined as P < 0.05.

Granulocytes, monocytes and lymphocytes were present after both accutase (Step 1) and collagenase (after mechanical dissociation, Step 2; Fig. 4A) enzymatic digestion. T cells, tissue-resident T cells (CD103^+^), lymphocytes and CD45^+^ cells had greater median proportions of single cells following Step 2 but were not significantly increased. For CD45^−^ cells that expressed markers associated with non-immune cells (Fig. 4B), endothelial (CD31^+^) cells demonstrated similar frequencies between Steps 1 and 2. Fibroblast (CD90^+^) cells were found in significantly greater proportion following Step 2 disaggregation.

Single cells recovered from Step 2 had a significantly greater proportion of granulocytes, monocytes and CD45^−^ cells that were dead, and a similar trend was seen for lymphocytes (Fig. 4C). Despite the increased cell death, the greater proportion of tissue-resident T cells and fibroblast cells recovered following Step 2 suggest the addition of mechanical disaggregation, and collagenase can recover more embedded immune and non-immune cells for further analysis, similar to previously published observations [19]. Following Step 1, 2.8 × 10^6^ single cells (median) were recovered from 5 g of myometrium tissue (Fig. 4D). From the remaining tissue that was then processed in Step 2, 1.5 × 10^6^ single cells (median) were recovered. Both steps combined yielded a median total of 3.9 × 10^6^ single cells from 5 g of myometrium tissue. When using this protocol, to identify if protocol Step 2 will induce changes in assay outcomes, we would recommend comparing cell subset phenotype and function between cells extracted from myometrium using accutase alone, and those with collagenase and mechanical disaggregation.

In order to determine which mechanical digestion protocol was better for isolation of viable cells at high yield, the ‘(Mouse) Adult Heart’ (AH, ‘m_adult_heart_01.01’; 16 s × 1 cycle) preset programme was previously tested against the NH setting. A lower proportion of neutrophils, granulocytes, monocytes and fibroblasts were detected by flow cytometry when the AH programme was used (Supplementary Fig. 1); percent viability was detrimentally affected, and median single-cell yield was greater for the NH than for AH programme (1.3 × 10^6^ and 6.6 × 10^5^, respectively).

### Comparison of cell frequencies from uterine tissues

**Figure 5.**
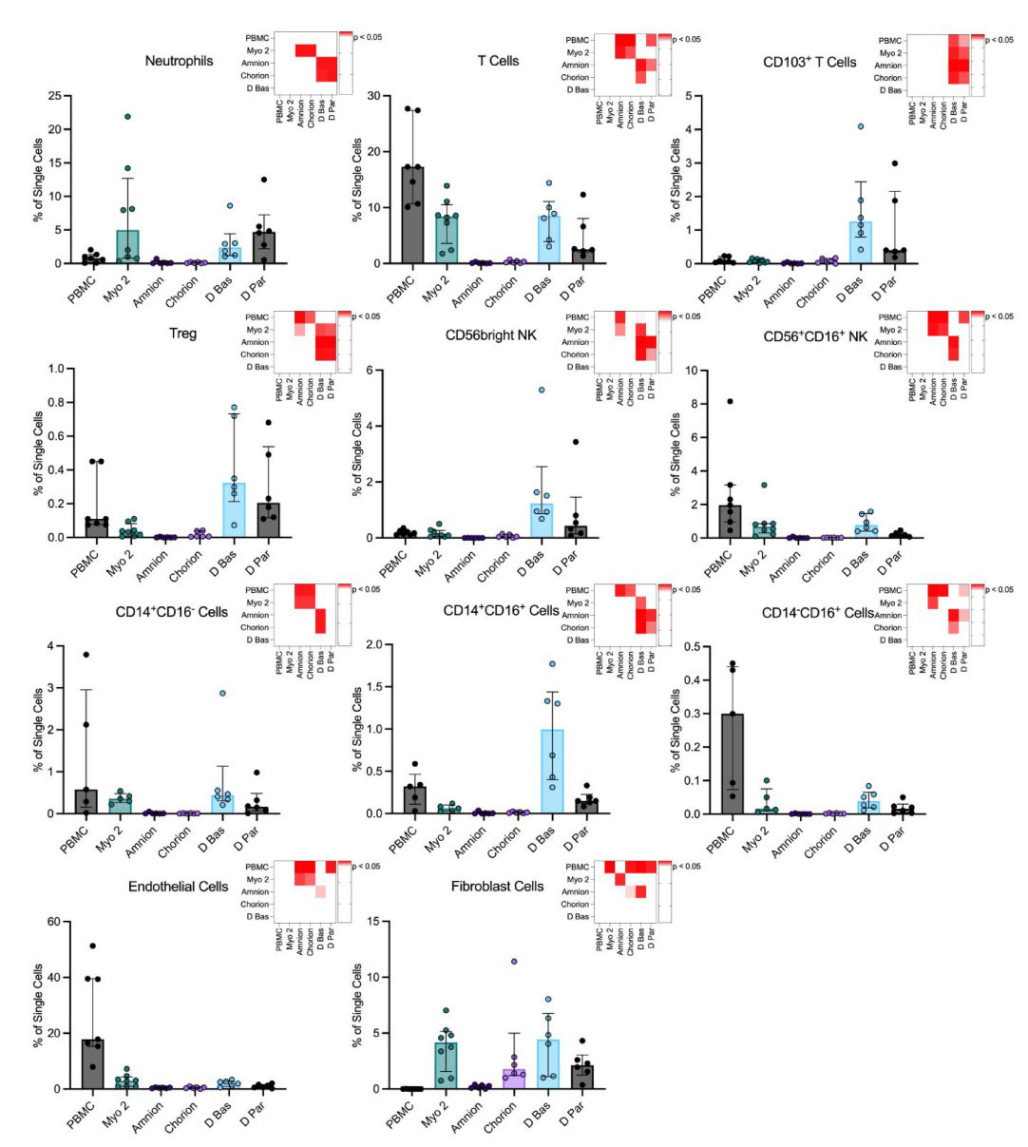
Comparison of single cells from PBMC and uterine tissues. Frequency of cell populations in the single cells retrieved from uterine tissues: myometrium tissues after disaggregation with accutase, collagenases and mechanical disaggregation (Myo 2; *N* = 8), amnion (*N* = 6), chorion (*N* = 6), decidua basalis (Dec Bas; *N* = 6) and decidua parietalis (Dec Par; *N* = 6). PBMC frequencies (*N* = 7) have been included for comparison. The heatmaps show where significant differences, indicated by red colour intensity, were seen between tissues. Multiple comparisons used to compare tissue types were undertaken using Kruskal–Wallis test corrected for false discovery rate and significance defined as *P* < 0.05.

We compared the constituent populations of recovered single cells from uterine tissues disaggregated using both protocols (Fig. 5 and Table 1). Significantly lower frequencies of neutrophils and T cells in both amnion and chorion were found compared to myometrium and decidua tissues. Tissue-resident T cells, regulatory T cells and CD56bright NK cells demonstrated significantly greater frequencies in decidua tissues than in the myometrium, amnion and chorion. CD56^+^CD16^+^ NK cells and endothelial cells were found at significantly higher proportions in the myometrium than in both amnion and chorion. CD14^+^CD16^−^, CD14^+^CD16^+^ and CD14^−^CD16^+^ monocyte-sized cells were found at significantly higher proportions in myometrium and decidua basalis tissues than in amnion and chorion. Fibroblast cells were found at significantly higher frequencies in myometrium, chorion and decidua basalis than in amnion.

**Table 1.** Summary of typical uterine tissue single-cell counts, viability, immune cell and non-immune cell frequencies obtained from Protocols 1 and 2

Tissue	Myo 1	Myo 2	Amnion	Chorion	Dec Bas	Dec Par
Single-cell counts/5 g						
Median (×10^5^)	28	15	45	20	43	95
Range (×10^5^)	4–90	6–18	7–187	4–70	28–59	45–118
Granulocytes						
Viability (Live %) median	89.5	65.6	35.1	14.7	88.3	86.9
%single cells median	7.3	7.4	0.1	0.1	2.8	5.0
%single cells range	1.5–18.1	2.0–27.1	0.04–1.5	0.07–0.26	1.3–9.2	0.7–12.7
Lymphocytes						
Viability (Live %) median	97.0	92.9	97.3	89.5	98.9	99.4
%single cells median	7.6	15.9	0.1	0.5	14.9	4.5
%single cells range	2.6–35.1	3.4–24.1	0.02–0.6	0.1–1.0	7.8–27.3	2.6–21.5
Monocytes						
Viability (Live %) median	95.0	76.8	80.0	38.9	93.2	93.7
%single cells median	3.4	3.3	0.02	0.06	2.2	0.9
%single cells range	1.0–9.8	0.8–8.8	0.006–0.2	0.04–0.1	1.1–5.2	0.4–2.5
CD45^−^ cells						
Viability (Live %) median	99.7	98.1	81.5	93.4	98.9	98.9
%single cells median	72.1	64.1	97.3	88.1	60.2	68.4
%single cells range	30.8–92.3	26.0–83.4	69.5–99.2	70.1–94.3	49.1–73.2	56.0–76.6
Neutrophils						
%single cells median	3.8	5.0	0.07	0.1	2.4	4.7
%single cells range	0.4–10.3	0.4–21.9	0.03–0.7	0.04–0.2	1.1–8.6	0.5–12.5
T cells						
%single cells median	4.0	8.4	0.07	0.3	8.5	2.5
%single cells range	1.4–18.3	1.8–13.9	0.0009–0.3	0.06–0.7	3.1–14.4	1.3–12.3
Treg						
%single cells median	0.02	0.04	0.0006	0.007	0.5	0.2
%single cells range	0.01–0.1	0.007–0.1	0.0–0.005	0.005–0.04	0.07–0.8	0.1–0.7
CD103^+^ T cells						
%single cells median	0.02	0.06	0.009	0.05	1.3	0.4
%single cells range	0.002–0.09	0.02–0.2	0.0002–0.05	0.002–0.2	0.4–4.1	0.2–3.0
CD56bright NK						
%single cells median	0.2	0.1	0.0005	0.05	1.2	0.4
%single cells range	0.03–0.2	0.04–0.5	0.0–0.008	0.009–0.1	0.7–5.3	0.08–3.4
CD56^+^CD16^+^ NK						
%single cells median	0.4	0.7	0.007	0.02	0.8	0.2
%single cells range	0.2–3.4	0.1–3.2	0.001–0.8	0.007–0.03	0.4–1.6	0.07–0.5
CD14^+^CD16^−^ monocyte-sized cells						
%single cells median	0.5	0.4	0.009	0.008	0.4	0.2
%single cells range	0.05–1.7	0.2–0.5	0.0002–0.5	0.005–0.02	0.2–2.9	0.01–1.0
CD14^+^CD16^+^ monocyte-sized cells						
%single cells median	0.4	0.06	0.002	0.01	1.0	0.2
%single cells range	0.03–0.7	0.02–0.1	0.0–0.04	0.004–0.02	0.3–1.8	0.08–0.3
CD14^−^CD16^+^ monocyte-sized cells						
%single cells median	0.2	0.02	0.0	0.001	0.04	0.02
%single cells range	0.1–0.5	0.006–0.1	0.0–0.003	0.001–0.004	0.01–0.08	0.003–0.05
Endothelial cells						
%single cells median	3.6	3.0	0.5	0.5	2.3	0.9
%single cells range	0.5–16.7	0.9–7.2	0.3–0.6	0.09–1.0	0.6–3.3	0.2–2.1
Fibroblast cells						
%single cells median	1.4	4.2	0.2	1.8	4.4	2.1
%single cells range	0.2–6.2	0.8–7.0	0.01–0.4	1.0–11.4	1.0–8.0	0.4–4.3

Myo 1, myometrium tissues after disaggregation with accutase alone; Myo 2, same as Myo 1 but followed by collagenase and mechanical disaggregation; Dec Bas, decidua basalis; Dec Par, decidua parietalis.

Identifying the interactions between cells that occur prior to and during physiological labour is essential to understanding how these mechanisms are disrupted to cause labour-related pathologies. As we have demonstrated, single cells recovered from our protocols can be phenotyped using flow cytometry. As surface marker expression is maintained, fluorescence-activated cell sorting or antibody-conjugated magnetic beads can be used to isolate specific subsets for primary culture or functional assays. We have previously published an investigation into decidual NK cells, isolated with the protocols detailed here, using the extracted cells to identify phenotypic changes across the human reproductive cycle, and differences in cell degranulation, IFNγ, IL-8, TNFα and granulocyte-macrophage colony stimulating factor (GM-CSF) responses to phorbol-12-myristate-13-acetate (PMA) and ionomycin stimulation [11]. Additionally, single-cell RNA-sequencing methods are improving our understanding of transcriptional heterogeneity between cell subsets and clinical outcomes, and applying this method to cells derived using our protocols would allow the investigation of both immunological and non-immune cell transcriptomes.

## Supplementary Material

iqac010_supplementary_data

## Data Availability

The data underlying this article are available in the article and in its online supplementary material.
