# Supplementary material for: Isolation of single cells from human uterus in the third trimester of pregnancy: myometrium, decidua, amnion and chorion
Source: Oxf Open Immunol. 2022 Nov 23;3(1):iqac010. doi: 10.1093/oxfimm/iqac010 (PMC9914580; doi:10.1093/oxfimm/iqac010)
Supplement: iqac010_supplementary_data [file iqac010_supplementary_data.pdf]

Supplementary Figures

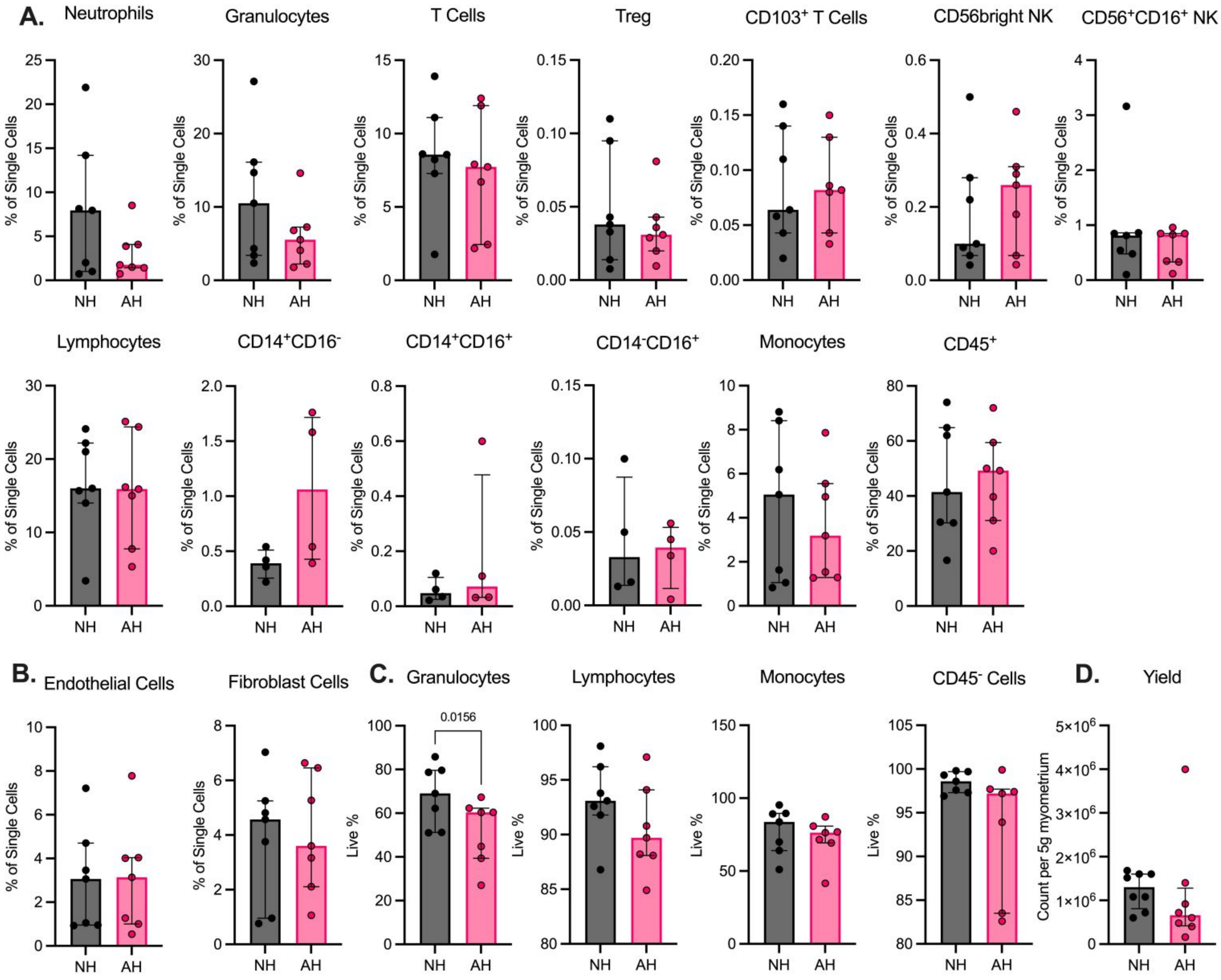

Supplementary Figure 1 – Comparison of gentleMACS neonatal heart versus adult heart disaggregation setting

Comparison of cells recovered from Step 2 that were mechanically disaggregated using either the gentleMACS neonatal heart (NH) or adult heart (AH; N=7) setting. **A.** show the frequency of immune cell subsets within the single cells gate. **B.** show the frequency of endothelial and fibroblast cells within the single cell gate. **C.** show the proportion of live cells in leukocyte and CD45$^-$ cell populations. **D.** shows the recovered cell counts per 5g of myometrium (N=8). For **A.-D.**, paired non-parametric Wilcoxon tests were used and significance defined as $p < 0.05$.

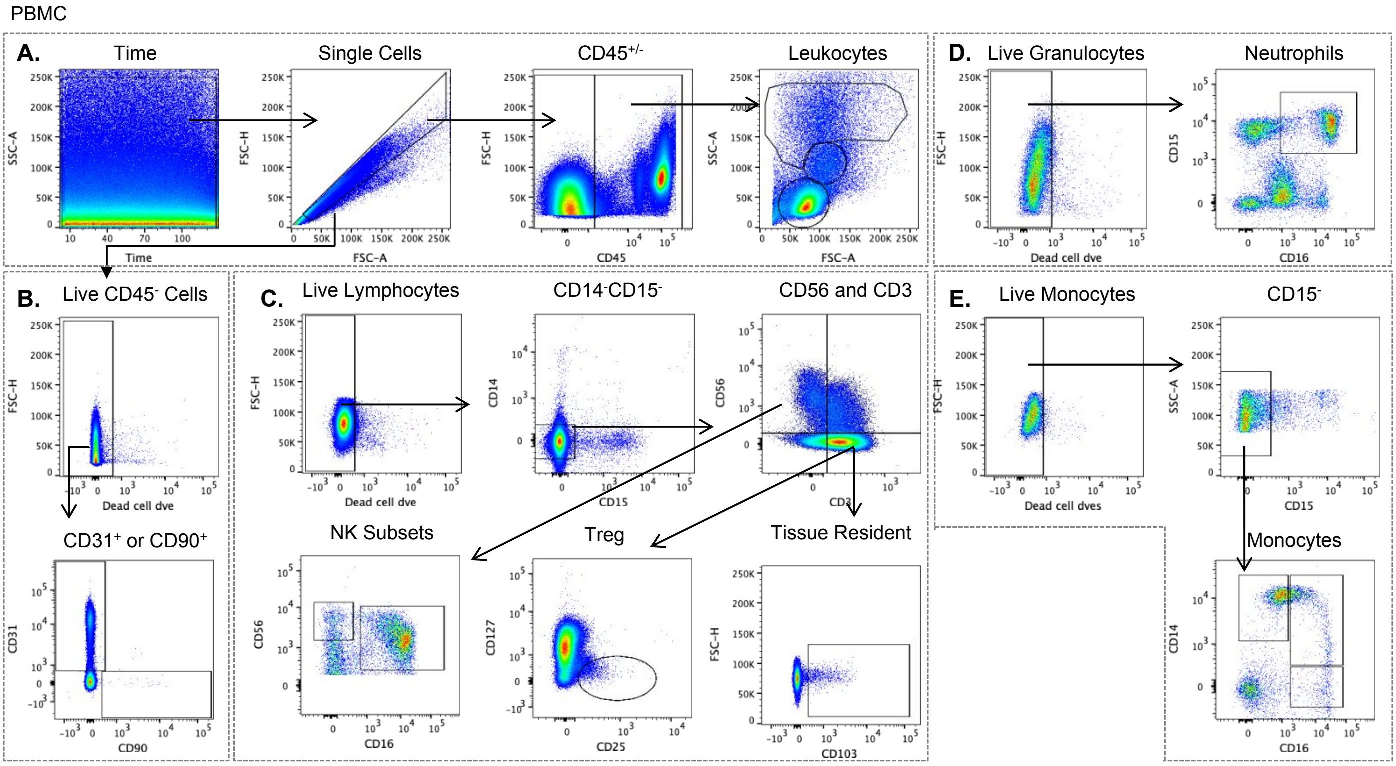

Supplementary Figure 2 – Fluorescent cytometry gating strategy of PBMC

PBMC were stained with the above panel of fluorescent monoclonal antibodies and acquired immediately on a BD Fortessa X20 cytometer. Samples were checked for any disrupted acquisition using a time gate, and any periods excluded prior to further gating. **A.** Identification of single cells, $CD45^{+/-}$ cells, and $CD45^{+}$ gates for granulocytes, monocytes and lymphocyte subsets based on their size and granularity. **B.** Gating of live $CD45^{-}$ cells, then identification of $CD31^{+}$ (endothelial) or $CD90^{+}$ (fibroblast) cells. **C.** Gating of live lymphocytes, exclusion of $CD14^{+}$ and $CD15^{+}$ cells, identification of $CD3^{+}$ T cells, tissue resident T cells ($CD103^{+}$), and regulatory T cells ($CD127^{low}CD25^{+}$), and both CD56bright ($CD56^{high}CD16^{-}$) and $CD56^{+}CD16^{+}$ NK cell subsets. **D.** Gating of live granulocytes and identification of neutrophils as $CD15^{+}CD16^{+}$. **E.** Gating of live monocyte sized $CD15^{-}$ cells as $CD14^{+}CD16^{-}$, $CD14^{+}CD16^{+}$, and $CD14^{-}CD16^{+}$ subsets. Gates were set using both unstained and fluorescence minus one controls.

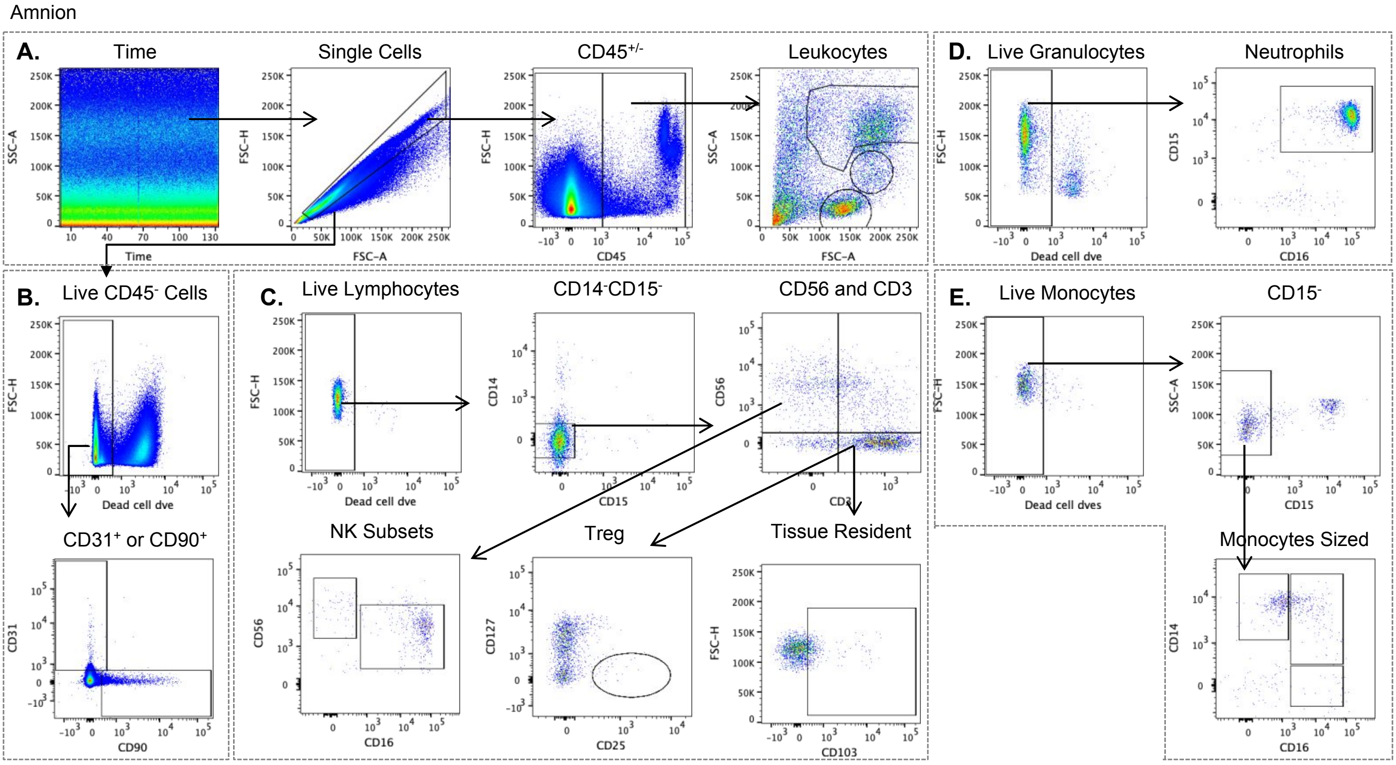

Supplementary Figure 3 – Fluorescent cytometry gating strategy of Amnion

Cells isolated from amnion tissue were stained with the above panel of fluorescent monoclonal antibodies and acquired immediately on a BD Fortessa X20 cytometer. Samples were checked for any disrupted acquisition using a time gate, and any periods excluded prior to further gating. **A.** Identification of single cells, $CD45^{+/-}$ cells, and $CD45^{+}$ gates for granulocytes, monocytes and lymphocyte subsets based on their size and granularity. **B.** Gating of live $CD45^{-}$ cells, then identification of $CD31^{+}$ (endothelial) or $CD90^{+}$ (fibroblast) cells. **C.** Gating of live lymphocytes, exclusion of $CD14^{+}$ and $CD15^{+}$ cells, identification of $CD3^{+}$ T cells, tissue resident T cells ($CD103^{+}$), and regulatory T cells ($CD127^{low}CD25^{+}$), and both CD56bright ($CD56^{high}CD16^{-}$) and $CD56^{+}CD16^{+}$ NK cell subsets. **D.** Gating of live granulocytes and identification of neutrophils as $CD15^{+}CD16^{+}$. **E.** Gating of live monocyte sized $CD15^{-}$ cells as $CD14^{+}CD16^{-}$, $CD14^{+}CD16^{+}$, and $CD14^{-}CD16^{+}$ subsets. Gates were set using both unstained and fluorescence minus one controls.

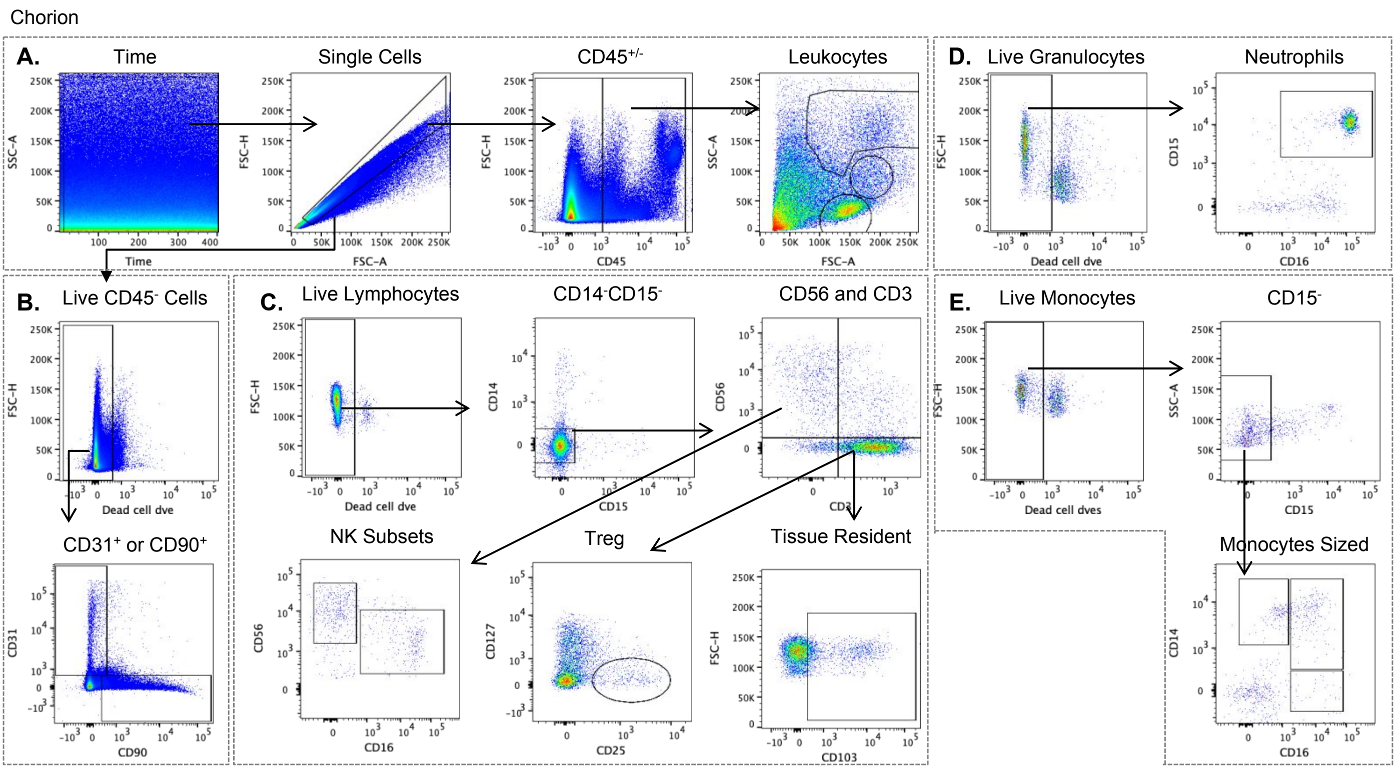

Supplementary Figure 4 – Fluorescent cytometry gating strategy of Chorion

Cells isolated from chorion tissue were stained with the above panel of fluorescent monoclonal antibodies and acquired immediately on a BD Fortessa X20 cytometer. Samples were checked for any disrupted acquisition using a time gate, and any periods excluded prior to further gating. **A.** Identification of single cells, CD45$^{+/-}$ cells, and CD45^{+} gates for granulocytes, monocytes and lymphocyte subsets based on their size and granularity. **B.** Gating of live CD45^{-} cells, then identification of CD31^{+} (endothelial) or CD90^{+} (fibroblast) cells. **C.** Gating of live lymphocytes, exclusion of CD14^{+} and CD15^{+} cells, identification of CD3^{+} T cells, tissue resident T cells (CD103^{+}), and regulatory T cells (CD127^{low}CD25^{+}), and both CD56bright (CD56^{high}CD16^{-}) and CD56^{+}CD16^{+} NK cell subsets. **D.** Gating of live granulocytes and identification of neutrophils as CD15^{+}CD16^{+}. **E.** Gating of live monocyte sized CD15^{-} cells as CD14^{+}CD16^{-}, CD14^{+}CD16^{+}, and CD14^{-}CD16^{+} subsets. Gates were set using both unstained and fluorescence minus one controls.

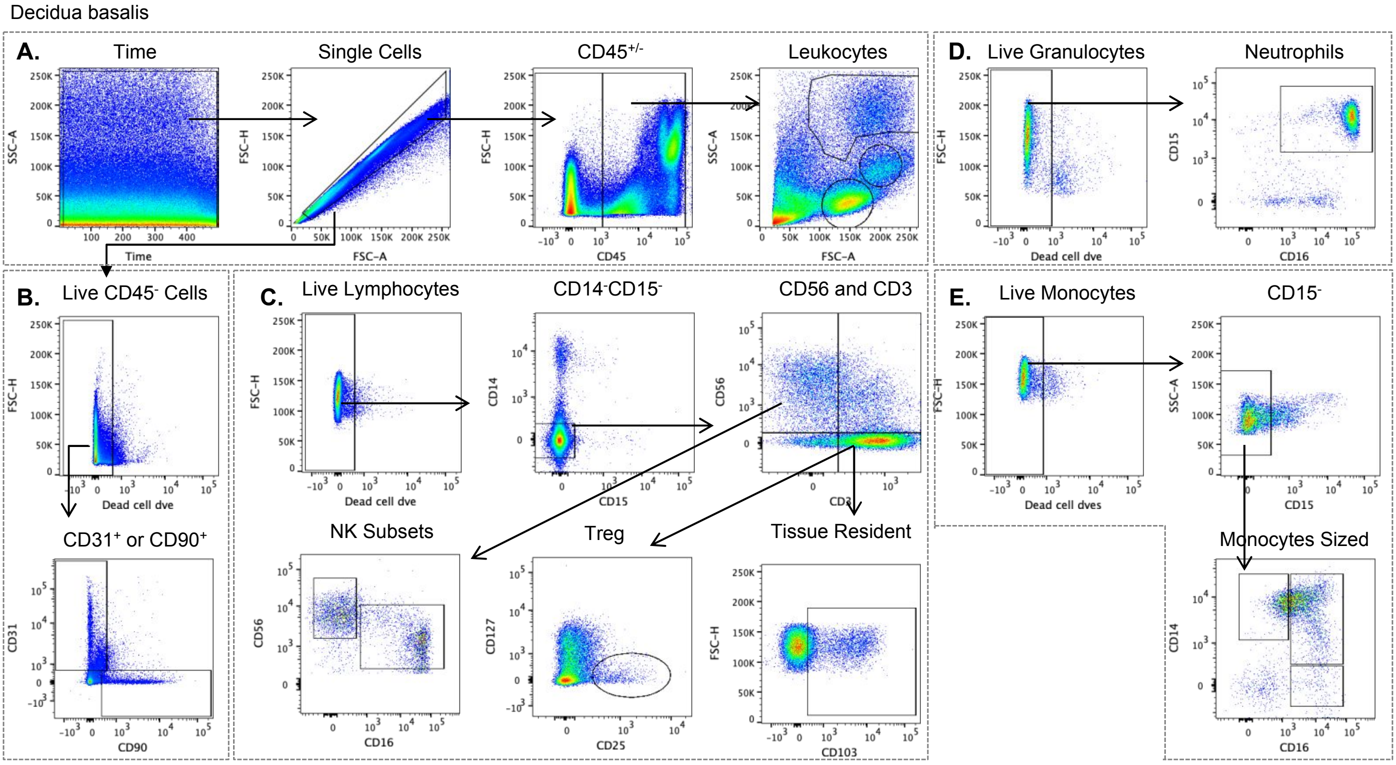

Supplementary Figure 5 – Fluorescent cytometry gating strategy of Decidua Basalis

Cells isolated from decidua basalis tissue were stained with the above panel of fluorescent monoclonal antibodies and acquired immediately on a BD Fortessa X20 cytometer. Samples were checked for any disrupted acquisition using a time gate, and any periods excluded prior to further gating. **A.** Identification of single cells, $CD45^{+/-}$ cells, and $CD45^{+}$ gates for granulocytes, monocytes and lymphocyte subsets based on their size and granularity. **B.** Gating of live $CD45^{-}$ cells, then identification of $CD31^{+}$ (endothelial) or $CD90^{+}$ (fibroblast) cells. **C.** Gating of live lymphocytes, exclusion of $CD14^{+}$ and $CD15^{+}$ cells, identification of $CD3^{+}$ T cells, tissue resident T cells ($CD103^{+}$), and regulatory T cells ($CD127^{low}CD25^{+}$), and both CD56bright ($CD56^{high}CD16^{-}$) and $CD56^{+}CD16^{+}$ NK cell subsets. **D.** Gating of live granulocytes and identification of neutrophils as $CD15^{+}CD16^{+}$. **E.** Gating of live monocyte sized $CD15^{-}$ cells as $CD14^{+}CD16^{-}$, $CD14^{+}CD16^{+}$, and $CD14^{-}CD16^{+}$ subsets. Gates were set using both unstained and fluorescence minus one controls.

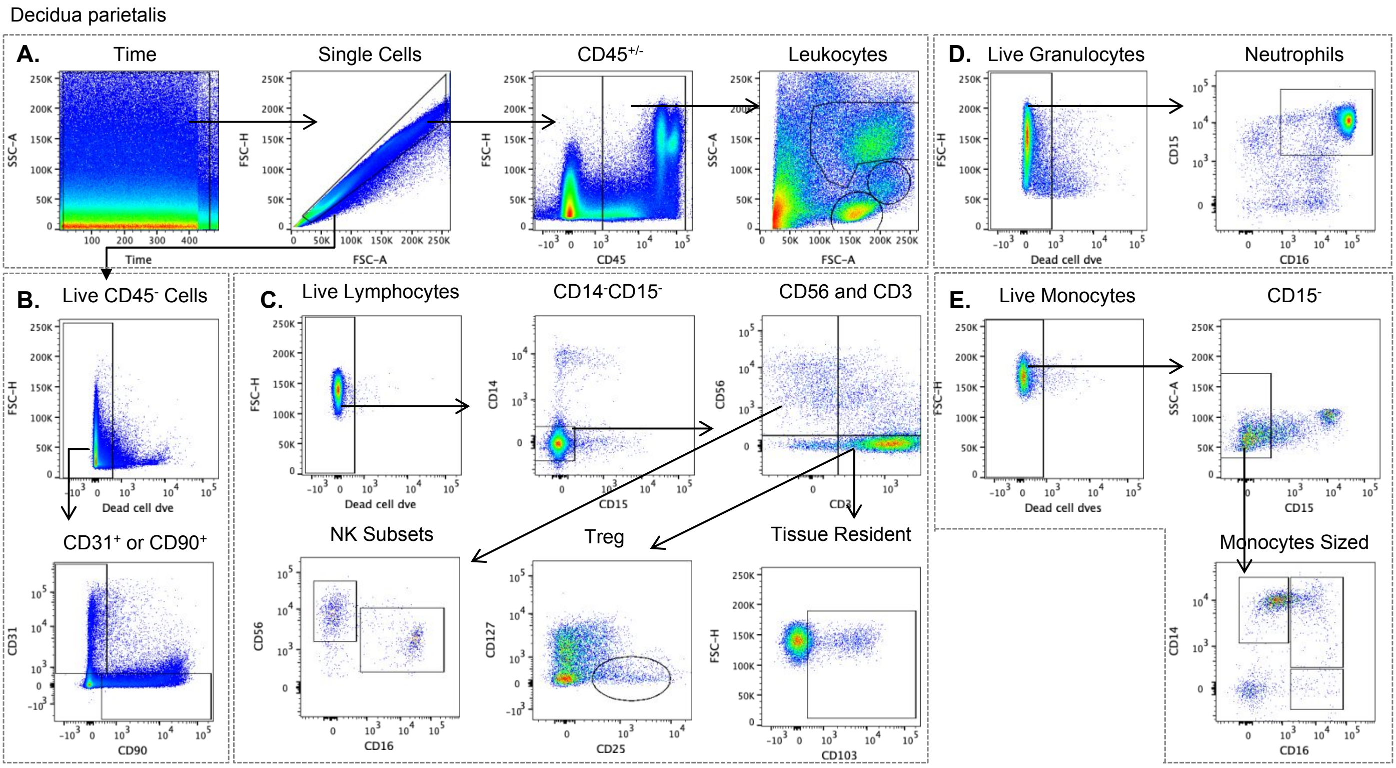

Supplementary Figure 6 – Fluorescent cytometry gating strategy of Decidua Parietalis

Cells isolated from decidua parietalis tissue were stained with the above panel of fluorescent monoclonal antibodies and acquired immediately on a BD Fortessa X20 cytometer. Samples were checked for any disrupted acquisition using a time gate, and any periods excluded prior to further gating. **A.** Identification of single cells, $CD45^{+/-}$ cells, and $CD45^{+}$ gates for granulocytes, monocytes and lymphocyte subsets based on their size and granularity. **B.** Gating of live $CD45^{-}$ cells, then identification of $CD31^{+}$ (endothelial) or $CD90^{+}$ (fibroblast) cells. **C.** Gating of live lymphocytes, exclusion of $CD14^{+}$ and $CD15^{+}$ cells, identification of $CD3^{+}$ T cells, tissue resident T cells ($CD103^{+}$), and regulatory T cells ($CD127^{low}CD25^{+}$), and both CD56bright ($CD56^{high}CD16^{-}$) and $CD56^{+}CD16^{+}$ NK cell subsets. **D.** Gating of live granulocytes and identification of neutrophils as $CD15^{+}CD16^{+}$. **E.** Gating of live monocyte sized $CD15^{-}$ cells as $CD14^{+}CD16^{-}$, $CD14^{+}CD16^{+}$, and $CD14^{-}CD16^{+}$ subsets. Gates were set using both unstained and fluorescence minus one controls.
